# Supplementary material for: Mechanisms governing the pioneering and redistribution capabilities of the non-classical pioneer PU.1
Source: Nat Commun. 2020 Jan 21;11:402. doi: 10.1038/s41467-019-13960-2 (PMC6972792; doi:10.1038/s41467-019-13960-2)
Supplement: Supplementary file 7 — Source data [file 41467_2019_13960_MOESM7_ESM.zip › Source_Data/FigureS2/FigureS2B_MotifScanOutput/KmeansCluster6/homerResults/motif37.similar.html]

motif37

## Information for motif37

A
G
C
T
A
C
G
T
C
G
A
T
G
A
C
T
G
C
T
A
C
T
G
A
C
G
T
A
C
T
G
A
A
G
T
C
A
G
T
C
C
G
T
A
C
G
A
T
A
T
G
C
  
Reverse Opposite:  

T
A
C
G
G
C
T
A
A
C
G
T
A
C
T
G
A
C
T
G
A
G
C
T
G
C
A
T
G
A
C
T
C
G
A
T
C
T
G
A
C
G
T
A
T
G
C
A
C
T
G
A
  

|  |  |
| --- | --- |
| p-value: | 1e-30 |
| log p-value: | -7.004e+01 |
| Information Content per bp: | 1.722 |
| Number of Target Sequences with motif | 83.0 |
| Percentage of Target Sequences with motif | 2.52% |
| Number of Background Sequences with motif | 187.8 |
| Percentage of Background Sequences with motif | 0.44% |
| Average Position of motif in Targets | 101.9 +/- 37.1bp |
| Average Position of motif in Background | 95.6 +/- 69.4bp |
| Strand Bias (log2 ratio + to - strand density) | 0.0 |
| Multiplicity (# of sites on avg that occur together) | 1.00 |
| Motif File: | file (matrix) reverse opposite |

### Similar de novo motifs found

|  |  |  |  |  |  |  |  |
| --- | --- | --- | --- | --- | --- | --- | --- |
| Rank | Match Score | Redundant Motif | P-value | log P-value | % of Targets | % of Background | Motif file |
| 1 | 0.785 | G C A T G T C A C G T A C T G A G T A C G T A C C G T A G A C T A G T C C G T A C T A G | 1e-27 | -64.088016 | 2.19% | 0.35% | motif file (matrix) |
| 2 | 0.825 | A C G T T C A G C T G A G A C T A C T G A C T G G A C T G A C T G A C T C A G T G T C A G C A T | 1e-18 | -43.450940 | 1.61% | 0.29% | motif file (matrix) |
| 3 | 0.878 | A G C T T C G A C T G A T C G A C T G A A G T C A T G C C T G A C G A T A T G C | 1e-14 | -34.426293 | 3.25% | 1.29% | motif file (matrix) |
